# A multilevel model of success goal orientation, risk aversion orientation, and creativity: the moderating effects of psychological resilience and trade union participation atmosphere

**DOI:** 10.3389/fpsyg.2025.1653545

**Published:** 2025-12-05

**Authors:** Wenjing Ke, Bingjie Sun

**Affiliations:** 1 Wuyi University, Wuyishan, China; 2 Xinjiang University of Science and Technology, Korla, China

**Keywords:** creativity, trade union participation atmosphere, achievement goal orientation, psychological resilience, multilevel model

## Abstract

Based on social cognitive theory, this study constructs and validates a cross-level theoretical model through three waves of longitudinal surveys, revealing the mechanism by which union participation climate influences employee creativity through psychological resilience. Analysis of a sample of 383 employees from 77 teams shows that union support climate plays a key moderating role in the relationship between success goal orientation and creativity. When employees perceive higher union support, the indirect effect of success goal orientation on creativity, mediated by psychological resilience, is significantly enhanced (E = 0.09, SE = 0.05, *p* < 0.05). In contrast, in low-support climates, this indirect effect is markedly weakened (E = 0.02, SE = 0.01, *p* > 0.05). These findings have significant implications for organizational management practices: establishing supportive mechanisms for union participation can enhance employees’ psychological safety, thereby promoting innovative behaviours. At the same time, excessive control should be avoided to prevent stimulating employees’ risk-averse tendencies.

## 1 Introduction

Against the backdrop of accelerated globalization and technological progress, market environments and competition are increasingly characterized by volatility (Carson et al., 2012), uncertainty (Dreyer and Grønhaug, 2004), complexity (Schuh et al., 2019), and ambiguity (Carson et al., 2012). In this context, creativity has become a key driving force for the survival and development of enterprises (Bousinakis and Halkos, 2021). According to the Global Creativity Index report, creative enterprises have a significantly higher average annual revenue growth rate than the industry average (Index, 2011). Creativity arises from the combined influence of individual, motivational, and contextual factors; yet, existing research tends to focus on independent analyses of individual or environmental characteristics, lacking an integrated perspective (Bousinakis and Halkos, 2021).

To this end, our findings, along with those of Anderson et al. (2014) and colleagues, integrate frameworks to expand the understanding of creativity by examining personal and environmental characteristics in isolation. Through examining personal and environmental characteristics in isolation. Extend multi-layered frameworks of social cognitive theory, achievement goal theory, and creativity theory to explore how individual characteristics and informal organizational relationships jointly stimulate achievement motivation, thereby promoting employees’ union creativity. Among these, employee creativity, as the starting point of the creative process (Lubart, 2001), is the focus of analysis. We particularly focus on the employee union as the unit of analysis, distinguishing between (Wallace et al., 2016) he individual perception level (such as employees’ cognitive understanding of union support) and the aggregate level (such as the overall innovative atmosphere of the union).

**Figure 1:**
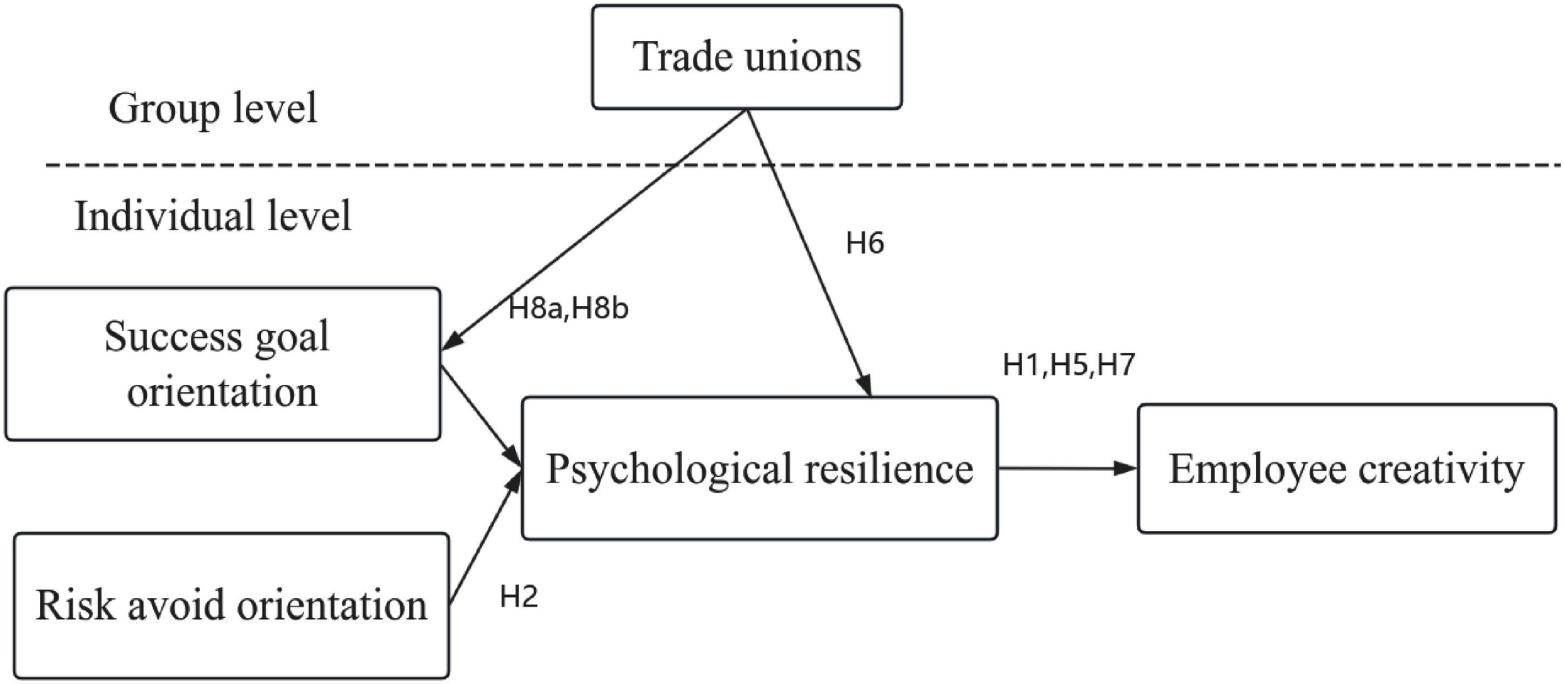
Hypothesised moderated mediation model. All hypothesized relationships are positive except for H3 and H5, which are negative. H4, H5, and H7 represent indirect effects. H8a and H8b together represent a cross-level, first-stage moderated mediation effect. H = hypothesis.

At the individual level, we focus on the impact of achievement goal orientation (including achievement motivation and risk-avoidance motivation) on creativity. At the organizational level, we examine how informal individual-organizational interactions support goal-oriented and creative processes (Eschenbächer and Zarvić, 2012). As shown in Figure 1, psychological resilience mediates the relationship between achievement goal orientation and union creativity (DeShon and Gillespie, 2005). Psychological resilience encompasses active cognition, emotional regulation, and coping abilities in the face of adversity (Hartwig et al., 2020), and it satisfies the needs for autonomy, competence, and relatedness as outlined in social cognitive theory (Bussey and Bandura, 1999). The fulfilment of these needs helps enhance union creativity, job satisfaction, and organizational commitment (Parent and Séguin, 2007).

This study proposes that when employees possess a goal orientation for participation in creation (achievement goal orientation) and are in a supportive union environment (such as an informal relationship atmosphere in highly unionised organisations), creativity opportunities will significantly increase. We adopt a multilevel research approach, integrating contextual and individual factors (Bandura, 2001), and utilize Self-Determination Theory (SDT) to explain the synergistic effects of these factors. Furthermore, we extend the indirect influence pathways of unions on creativity (Teixeira et al., 2012). Cunningham (2010) notes that union characteristics enhance psychological resilience by meeting basic psychological needs, thereby promoting creativity. The study also explores the mediating mechanism of psychological resilience between individual and group effects, addressing the gap in existing research regarding its operational mechanisms.

## 2 Theoretical background and hypotheses

In today’s rapidly evolving business landscape, creativity serves as the driving force behind both enterprise development and trade union innovation (Menezes-Filho and Van Reenen, 2003). Psychological resilience enables a trade union participation atmosphere to navigate creativity-related frustrations effectively (Yongti and Ling, 2018). Moreover, the organizational environment cultivated by a trade union participation atmosphere significantly influences their creative processes, warranting in-depth examination (Hampton, 2015). This competitive advantage continuously evolves within knowledge-driven market contexts, amid enterprises’ ongoing innovation cycles, with trade union participation and creative contributions providing sustainable development pathways for organizations (Cunningham, 2010). Simultaneously, achievement goal orientation demonstrates significant relationships with psychological resilience, which subsequently shapes the creative motivation atmosphere in trade unions; consequently, psychological resilience functions as the driving force behind individual creative processes (Walia, 2019). Nevertheless, individual creativity limitations persist, while the trade union environment substantially impacts collective creative output (Hampton, 2015). Therefore, our subsequent chapter first examines the role of psychological resilience in trade union creativity, subsequently explicates the relationship between achievement goal orientation and psychological resilience, and ultimately highlights trade union involvement as a contextual variable influencing individual-level outcomes (Sutherland, 2024).

### 2.1 Achievement goal orientation and trade union and creativity

Goal orientation, defined as an individual’s or situational preference for achievement (Niemivirta et al., 2019), helps explain why organisations prioritise developing innovative solutions to address performance challenges in unionised environments (Townsend et al., 2001). As a situational variable (Burke et al., 1996), goal orientation interacts with values related to ideal self-evaluation (such as hopes and aspirations) to enhance individual focus and risk-averse motivation, thereby strengthening organizational commitment (Wowak and Hambrick, 2010). Although previous studies often viewed goal orientation as an individual trait within a tendency framework (Cellar et al., 2011), it manifests in both formal and informal organizational relationships. The formation of goal orientation depends on situational factors, such as psychological resilience and empowerment, which influence individuals’ goal adoption (Chadwick and Raver, 2015). Our research indicates that collective responses to adversity can be strengthened through shared situational exposure among group members, thereby transforming perceived psychological resilience into unique goal orientations (Ruzek, 2025). Therefore, team goal orientation is a product of group interaction (Huang et al., 2019). In this study, organizational goal orientation is defined as a workplace-centred achievement framework within union participation environments (Kay, 2015), acknowledging the complex relationship between unions, achievement goal orientation, and innovation outcomes (Harms et al., 2010). As organizations representing members’ rights and promoting their welfare, union participation environments exhibit three goal-oriented characteristics (Finnestrand, 2023): learning-oriented, performance-oriented, and performance-avoidance goals, all of which are manifested at the organizational level (Gong et al., 2017). Specifically, learning-oriented union members view unions as platforms for acquiring knowledge and enhancing skills, actively participating in training and knowledge-sharing activities to expand their capabilities (Illeris, 2003). Performance-oriented unions enhance individual performance by integrating organizational resources (Lee et al., 2009) and establishing transparent reward mechanisms and equitable task allocation systems through negotiation, thereby promoting excellence (Onavwie et al., 2023). In contrast, performance-avoidance unions prioritize maintaining job stability and preventing punitive outcomes (Padilla-Walker and Carlo, 2014), adopting flexible performance standards that allow for gradual improvement among members. From the perspective of exchange theory, these distinct goal orientations provide crucial foundations for evidence-based policy formulation and activity planning. Learning-oriented unions typically conduct vocational training and skill enhancement through expert lectures and peer knowledge sharing (Michailova and Hutchings, 2006). Performance-oriented unions actively engage in corporate dialogue and optimize incentive mechanisms to promote work performance through equitable incentives (Wang et al., 2025). Performance-avoiding unions advocate for establishing reasonable performance support systems to balance performance-enhancing requirements with operational sustainability (Manning and Watkins, 2013). Therefore, we propose:

*H1a: Success goal motivation has a positive effect on trade union participation atmosphere and creativity.*

*H1b: Avoidance failure motivation has a negative impact on trade union participation, atmosphere, and creativity.*

### 2.2 Achievement goal orientation and psychological resilience

Psychological resilience is an individual’s capacity to withstand work-related stress, while goal orientation is a stable yet adaptable personality trait that reflects task engagement (Fletcher and Sarkar, 2013). This enduring trait comprises two motivational dimensions: success orientation and risk aversion. A strong success orientation drives individuals to set challenging goals (Raetze et al., 2021), actively seek opportunities, expand their capabilities, and experiment with innovative approaches. Resilient individuals can translate their pursuit of success into actionable objectives (Schuh et al., 2019). Risk aversion initially manifests as avoidance behaviours, but when perceived as growth opportunities, these reactions ultimately become a developmental momentum, culminating in achievement satisfaction upon task completion (Kavčič and Bertoncelj, 2010). Moreover, values associated with “ideal” self-evaluation (such as aspirations and desires) enhance cognitive focus, while risk aversion strengthens organizational commitment (Vandewalle et al., 2019). Empirical studies demonstrate that a success orientation has a positive influence on work performance and creativity, whereas risk aversion may undermine external organizational relationships and hinder innovation (Latham and Braun, 2009). This study further examines the mediating role of psychological resilience and its behavioural mechanisms. Perez-Freije and Enkel (2007) noted that creativity is a crucial prerequisite for creativity. Bryson et al.’s (2004) research highlights that union members ‘civic engagement (i.e., purpose-driven workplace behaviours), psychological resilience, and success motivation are key factors in career development. In union participation environments, highly motivated members are more willing to take on challenging tasks, such as exceeding performance expectations, exploring alternatives, and assuming calculated risks, compared to those with lower success motivation, all of which correlate with higher levels of psychological resilience (Magnano et al., 2016). Success-oriented unions emphasize going beyond assigned duties, achieving a sense of accomplishment, and boosting collective morale (Johnson and Johnson, 2005). The successful completion of tasks further reinforces the motivational cycle. Studies show that a learning goal orientation effectively enhances achievement motivation, while success motivation also promotes learning processes (Li and Shieh, 2016; Lu et al., 2023). Professional skill development largely depends on social learning within professional networks, which stimulates employees’ participation in innovative practices and creates new experiences (Krushkov and Zayakova-Krushkova, 2024).

Consequently, success-oriented union members tend to view experimental challenges as opportunities, with bold explorers typically demonstrating stronger vitality and learning capabilities (VandeWalle, 2001). Through exploratory behaviour, union members not only create, process, and implement innovative solutions but also continually accumulate psychological and behavioural resources (Yan et al., 2002). Meanwhile, discovery learning enhances success motivation by exposing learners to diverse knowledge and skills (Lu et al., 2023). While innovative concepts and cutting-edge solutions can open new career pathways, they may also foster risk-averse tendencies, such as conservative adherence to social norms and avoidance behaviour, which can sometimes lead to counterproductive outcomes (Massey, 2020). For instance, in product development environments, high-risk-averse union members often resist adopting new materials or technologies (Vandenberghe et al., 2011). Conversely, exploratory learning helps expand professional networks and facilitates collaboration with cross-disciplinary experts and peers. Therefore, we propose:

*H2: Success motivation has a positive effect on psychological resilience.*

*H3: Risk-avoidance motivation impairs psychological resilience.*

Based on the above, the relationship between H 1 to H 2 and H 3, we propose the following additional hypotheses:

*H4: Success motivation has a positive indirect effect on creativity through psychological resilience.*

*H5: Risk aversion motivation has an adverse indirect effect on creativity through psychological resilience.*

### 2.3 The role of employee trade union

Trade unions play a vital motivational role by providing social support for network relationships (Ebbinghaus, 2002), fostering experiential motivation and social learning; however, opportunities for behavioural pursuit depend on the strength of these social networks. Consequently, the presence of union support positively influences motivational tendencies that align with the psychological resilience benefits of social cognitive theory (SCT) principles. Humans possess three core psychological needs: autonomy, competence, and relatedness. In environments with strong union participation and challenging demands, high levels of union involvement are associated with enhanced psychological resilience (Zhang et al., 2024). Social cognitive theory further distinguishes intrinsic motivation from extrinsic motivation, where autonomy-related motivation corresponds to psychological resilience through self-regulation mechanisms. Self-determination theory researchers, such as Ryan et al. (1992), suggest that extrinsic motivation manifests at different levels of internalization, ranging from complete external control to gradual integration of personal values and goals. This process has a profound impact on both psychological and behavioural outcomes. Therefore, a supportive social environment for unions cultivates positive psychological resilience.

However, it is worth noting that the study’s samples were drawn from two union organizations with similar work tasks and primarily operational roles, which means the findings may not be applicable to managerial or knowledge-intensive positions (Song and Sun, 2018). Moreover, not all competency-based work environments can meet the psychological needs of autonomy, care, and belonging associated with union participation (Hon, 2012). Sutherland (2024) emphasized that autonomy in union participation requires complete independence and an intrinsic interest in the activity itself. Therefore, meeting the basic psychological needs of union participation is essential for enhancing psychological resilience (Shao et al., 2023). Work environments related to union participation—whether at the organizational, departmental, or team level—involve entities with decision-making authority and operational capabilities, as well as those mobilizing resources through active knowledge transfer (Tapia, 2013). Such environments foster positive knowledge exchange, organizational commitment, favourable organizational climates, and enhanced reward structures (Chen et al., 2010). Union participation benefits from the positive effects of organizational identity, as managers who provide social responsibility and organizational support for work engagement can effectively reduce destructive behaviour. Therefore, we propose:

*Hypothesis 6: Employee trade union positively relates to psychological resilience.*

*Hypothesis 7: Employee trade union has a positive indirect effect on creativity via psychological resilience*

*Hypothesis 8a: Employee trade union moderates the positive relationship between success orientation and psychological resilience, such that the relationship becomes stronger as the employee trade union is higher.*

*Hypothesis 8b: The positive indirect effect of an employee trade union, mediated via psychological resilience, is moderated by the employee trade union, such that the indirect effect becomes stronger as the employee trade union is higher.*

## 3 Methods

### 3.1 Participants and procedure

One hundred different supervisors lead these working groups from multiple organizations in China, including machine operators engaged in diverse tasks such as equipment maintenance, assembly, and quality inspection. Although the requirements for creativity vary across these positions, the industry is actively pursuing practical improvements through creative generation and the implementation of new operational processes, technological applications, or management models. The study employed a three-wave longitudinal design. Initially, 400 individuals were invited, and 390 participated and completed the first wave (T1) survey, providing self-reported data on achievement goal orientation, union participation climate, and workplace creativity, with a response rate of 97.5%. Approximately 1 month later, the 390 participants completed the second wave (T2) psychological resilience assessment. For the third wave (T3), supervisors rated their subordinates’ creative performance; 77 supervisors participated, resulting in a valid supervisor data recovery rate of 77% (77 out of 100). Seven employees lacked supervisor rating data; however, they were ultimately matched with data from 383 employees (from 77 teams), and their supervisors were included in the final analysis. All research procedures were approved by the Institutional Review Board (IRB) of the respective universities or research institutions. Participants signed a written informed consent form before participating, clearly understanding the research purpose, involuntariness, confidentiality measures, and the right to withdraw at any time; in the final sample, 97.74% were male (n = 348), with an average age of 43.7 years (SD = 8.2), an average tenure of 5.6 years (SD = 7.8) in their current organization, and distributed across work groups consisting of 2 to 15 unions, with an average of 3.2 unions per team (SD = 3.9). The educational level was primarily a bachelor’s degree (*M* = 4.1, SD = 3.7, coded as 1 = high school or below, 4 = bachelor’s degree, 5 = master’s degree or above). All data collection and analysis were completed within a three-month period.

### 3.2 Measures

These measures were implemented over 3 months: employees first completed assessments of achievement goal orientation and union involvement at Time 1 (T1); approximately 1 month later (T2), the same participants underwent psychological resilience assessments; and at Time 3 (T3), their supervisors rated their subordinates’ creativity-related performance. All scales used Likert-type scoring, with specific measurement tools as follows.

#### 3.2.1 Employee trade union

The measurement of employees’ perceived union support was conducted using a 6-item scale adapted from the Organisational Support Scale developed by Shore et al. (1994), which assesses employees’ perceptions of the supportive atmosphere within their union. All scale items were self-reported by employees. Example items include “The union truly cares about my well-being” and “The union will provide help when I need it.” Scoring was done using a 5-point Likert scale, ranging from 1 = “Strongly Disagree” to 5 = “Strongly Agree.” Data analysis indicated that the scale had high reliability in this study: Cronbach’s = 0.91, 95% CI [0.88, 0.93]; McDonald’s = 0.92, 95% CI [0.90, 0.94].

#### 3.2.2 Achievement goal orientation

The Achievement Goal Orientation Scale is based on the 10-item, three-dimensional version developed by Duan and Bu (2017). This study adopted two subdimensions: learning/mastery goals (success-oriented) and performance-avoidance goals (risk-averse). Each dimension includes five items, measured using a 5-point Likert scale ranging from 1 = ‘Strongly Disagree’ to 5 = ‘Strongly Agree’. Example items include ‘I set goals to improve my own skills’ (success-oriented) and ‘I avoid tasks that might expose my lack of ability’ (risk-averse). The total scale has a Cronbach’s *α* of 0.89, with a 95% confidence interval of [0.86, 0.91]. The coefficients for the two subdimensions are 0.87 [0.83, 0.90] (success-oriented) and 0.85 [0.81, 0.88] (risk-averse), respectively. There are no reverse-scored items.

#### 3.2.3 Psychological resilience

The Psychological Resilience Scale adopted the Chinese revised version of the 10-item CD-RISC, developed by Cheng et al. (2020). It consists of 10 items, with example items including “I can adapt to change and maintain flexibility” (positively scored) and “I feel overwhelmed by problems” (reverse scored). A 5-point Likert scale is used, ranging from 1 = “Rarely” to 5 = “Almost always.” The total score is calculated by summing the scores of all items, with higher scores indicating higher levels of psychological resilience. In this study, Cronbach’s alpha was 0.93, with a 95% CI of [0.91, 0.95], and McDonald’s omega was 0.94, with a 95% CI of [0.92, 0.95]. Items 4, 6, and 8 are reverse-scored. The scale was confirmed to have two dimensions in the confirmatory factor analysis (CFA), named “Motivation” and “Health Status,” consistent with the scale description terminology.

#### 3.2.4 Creativity

This study employed the Employee Creativity Scale developed by Gong et al. (2009), which comprises seven items. The scale includes example items such as “This person’s work results are creative” (positive scoring), with no explicitly mentioned reverse-scored items in the search results. A 5-point Likert scale was used, ranging from 1 (“strongly disagree”) to 5 (“strongly agree”). Total scores were calculated by summing all item responses, where higher scores indicate greater creativity levels. The Cronbach’s α coefficient was 0.88 [95% CI (0.91, 0.95)], and McDonald’s *Ω* coefficient was 0.92 [95% CI (0.92, 0.95)]. No specific items in the search results were explicitly marked as reverse-scored. Confirmatory factor analysis (CFA) confirmed the scale’s single dimension, termed employee ceativity, consistent with the scale’s descriptive terminology.

#### 3.2.5 Control variables

In terms of control variables, this study included employees ‘age, education level, tenure, gender, and union activity participation, as these factors have been widely recognized as influencing creativity (Binnewies et al., 2008). Among them, union activity participation was measured using the China Enterprise Union Practice Scale developed by Li et al. (2019), which includes dimensions such as coordinating labour relations, caring for employees’ welfare, and organizing recreational activities, comprising a total of 13 items. Although factors such as tenure and gender may also affect creativity, the selection of control variables in this study was primarily based on variables explicitly confirmed in existing literature to ensure model simplicity and statistical power. Variables not included will be explored in the future.

### 3.3 Confirmatory factor analysis

This study employed confirmatory factor analysis (CFA) to examine the structural specificity of employee trade union participation atmosphere, achievement goal orientation, psychological resilience, and creativity. The measurement model incorporated employee trade union items at both individual (level 1) and group (level 2) levels, featuring random intercepts at each level (Drager and Hay, 2012). Due to dependencies, all other measures were modelled at the individual level. Following Sagone and De Caroli (2014), psychological resilience was specified as two correlated factors: motivation and wellness. The model fit was good: *χ*^2^ (532) = 1475.73, CFI = 0.91, TLI = 0.90, RMSEA = 0.07, SRMR (within-group) = 0.06, SRMR (between-group) = 0.06, GFI = 0.90. All factor loadings exceeded 0.76 and were statistically significant. The high correlation between motivation and wellness factors (0.87) was consistent with previous validation studies (Gagné et al., 2015). Given that motivation and wellness are two dimensions of a higher-order psychological resilience construct. The primary focus was on this overarching construct, where we aggregated their composite scores (after group-mean centring) to form a single psychometric measure of logical resilience. The results support the distinctiveness of the constructs at their intended levels of analysis and justify combining motivation and wellness into a unified resilience score for subsequent analyses.

### 3.4 Aggregation of employee trade union participation atmosphere

Based on the framework proposed by Bennink et al. (2015), we followed a three-step procedure to justify aggregating individual-level data to the work group level: sufficient within-group homogeneity, sufficient between-group heterogeneity, and the natural occurrence of clusters. Since the work groups were naturally formed, we employed statistical indices to verify adequate within-group homogeneity and between-group heterogeneity, thereby justifying the aggregation of employee union data.

To assess within-group homogeneity, we calculated the within-group agreement index (Rwg) using the approach outlined by Lüdtke and Robitzsch (2009). The results demonstrated strong within-group consistency across key variables, with mean Rwg values of 0.93 and 0.87 (ranging from 0.78 to 0.99 and 0.63 to 0.99, respectively), and median values of 0.89 and 0.85. Furthermore, intracranial correlation coefficients—ICC (1) and ICC (2)—were computed further to evaluate homogeneity and the reliability of group means (Gavaghan et al., 2000). The obtained ICC (1) value of 0.30 indicates that 30% of the variance in individual responses was attributable to workgroup membership. The ICC (2) value of 0.72, exceeding the conventional threshold of 0.70, confirms the reliability of group-level means (Bennink et al., 2015). A one-way ANOVA further revealed significant between-group variance, *F* (74, 302) = 3.05, *p* < 0.05. Collectively, these results provide strong statistical support for aggregating employee union data to the group level in subsequent analyses.

### 3.5 Analysis strategy

Based on the assumption that individual data are nested under teams or supervisors and involve cross-level relationships, we used Mplus 6.12 software. We conducted hypothesis testing using multilevel path analysis based on robust maximum likelihood estimation (MLR). The MLR estimation method provides robust standard errors and chi-square statistics, effectively handling non-normal distributions and missing data. This analytical approach adheres to the modelling guidelines for multilevel mediation models, allowing for the simultaneous estimation of level-1 covariances, random effects, indirect effects, and complex cross-level mediating paths. Compared to traditional stepwise regression methods or fragmented analytical approaches in some structural equation models, this integrated framework enables the simultaneous and complete estimation of all model parameters, thereby reducing potential biases that arise from stepwise or local estimation. To test hypotheses related to unions, we constructed simultaneously estimated multilevel regression models, a framework that has been successfully used to simulate conditional indirect effects across levels. This study integrates path analysis for mediation testing within a multilevel context, thereby addressing the limitations of focusing solely on multilevel regression or mediation analysis based on hierarchical linear models when dealing with complex path models. Consistent with our individual-level motivation theory model, all individual-level variables (except the outcome variable ‘employee creativity’) were group-mean centred to ensure that the estimated effects purely reflect within-group variation without being confounded by between-group differences. In terms of model specification, the key theoretical path from employee union involvement to creativity was specified as a random slope, allowing for variation across different teams and thereby enabling the examination of robustness and cross-situational generalization.

## 4 Results

### 4.1 Model and hypotheses testing

Table 1 presents the means, standard deviations, and bivariate correlation coefficients. Before testing the multilevel moderated mediation model, we first need to analyze the relationships between variables at the individual level. To this end, we followed the research method of Xiang and Wang (2015) to construct a path model where motivation orientation (achievement goal orientation and risk-averse orientation) exerts an indirect effect on creativity through individual development (X → M → Y), while also considering direct effects and the nested effects of individuals within teams/supervisors (i.e., incorporating random intercept and slope terms). Additionally, age, educational background, gender, and work duration were included as control variables in the model, exerting fixed effects on individual development and creativity. The purpose of this analysis is: (a) to test the significance of the direct and indirect effects of X on Y through M; (b) to evaluate the significance of variability in indirect effects at the second-level unit (i.e., random effects). To validate the necessity of conducting moderation analysis at Level 2 (Bacharach et al., 2008), we hypothesized that there are significant indirect effects and variability at this level. We thus adopted a multilevel moderated mediation analysis approach, taking into account the influence of employee participation atmosphere.

**Table 1:** Means, standard deviations, and bivariate correlations among study variables.

Variable	*M*	*SD*	1	2	3	4	5	6	7	8	9
1. Success orientation	3.73	0.92	0.07	0.09	0.15*	(0.89)	–				
2. Risk avoidance	3.21	0.78	−0.05	−0.07	−0.06	0.35*	(0.87)	–			
3. Psychological resilience	3.11	0.85	0.01	0.13*	0.8	0.39*	−0.11*	(0.91)	–		
4. Creativity	3.71	1.13	−0.02	−0.03	0.05	0.13*	−0.09	0.30*	(0.88)	–	
5. Employee Trade Union	3.09	1.12	0.01	0.15*	0.22*	0.15*	−0.15*	0.28*	49*	(0.91)	

*N* = 383. Employee trade union relations atmosphere measures factors such as employees’ expectations of the organization before joining a union. The diagonal values are the internal consistency reliability coefficients (Cronbach’s α) of each variable. *p < 0.05 indicates significance at the 0.05 level.

We propose that psychological resilience is related to creative activities (Hypothesis 1). Additionally, we suggest that success-oriented goal focus and risk-avoidance tendency are related to psychological resilience (Hypotheses 2 and 3). Furthermore, we hypothesize that psychological resilience plays a mediating role in the following relationships: (a) between success-oriented goal focus and creative activities (Hypothesis 4); (b) between risk-avoidance tendency and creativity (Hypothesis 5). Employee union membership is related to psychological resilience (Hypothesis 6), and the psychological resilience of the union atmosphere is related to creativity (Hypothesis 7). Additionally, employee union membership moderates the following relationships: (a) between success orientation and psychological resilience (Hypothesis 8a); (b) between success orientation, psychological resilience, and creativity (Hypothesis 8b).

The results of individual-level analyses support these hypothesized relationships. Specifically, path model results show that psychological resilience is positively correlated with creative activities (*γ* = 0.35, *p* < 0.01). Additionally, success goal orientation is positively correlated with psychological resilience (*γ* = 0.19, *p* < 0.01), whereas risk-averse orientation is negatively correlated with (*γ* = −0.07, *p* < 0.01) it. To test the indirect effects (Hypotheses 4 and 5), we employed a parametric self-service method to calculate the confidence interval of indirect effects through 20,000 Monte Carlo simulations. The results showed that success-oriented attention exerts a positive indirect effect on creative activities through psychological resilience (effect size = 0.05, 95% CI = 0.009–0.105); risk-averse attention also demonstrates a positive indirect effect through psychological resilience; whereas risk-avoidance oriented attention produces an adverse indirect effect on creative activities via psychological resilience (effect size = −0.03, 95% CI = −0.052 to −0.004). Overall, these findings provide strong support for Hypotheses 1 through 5.

**Figure 2:**
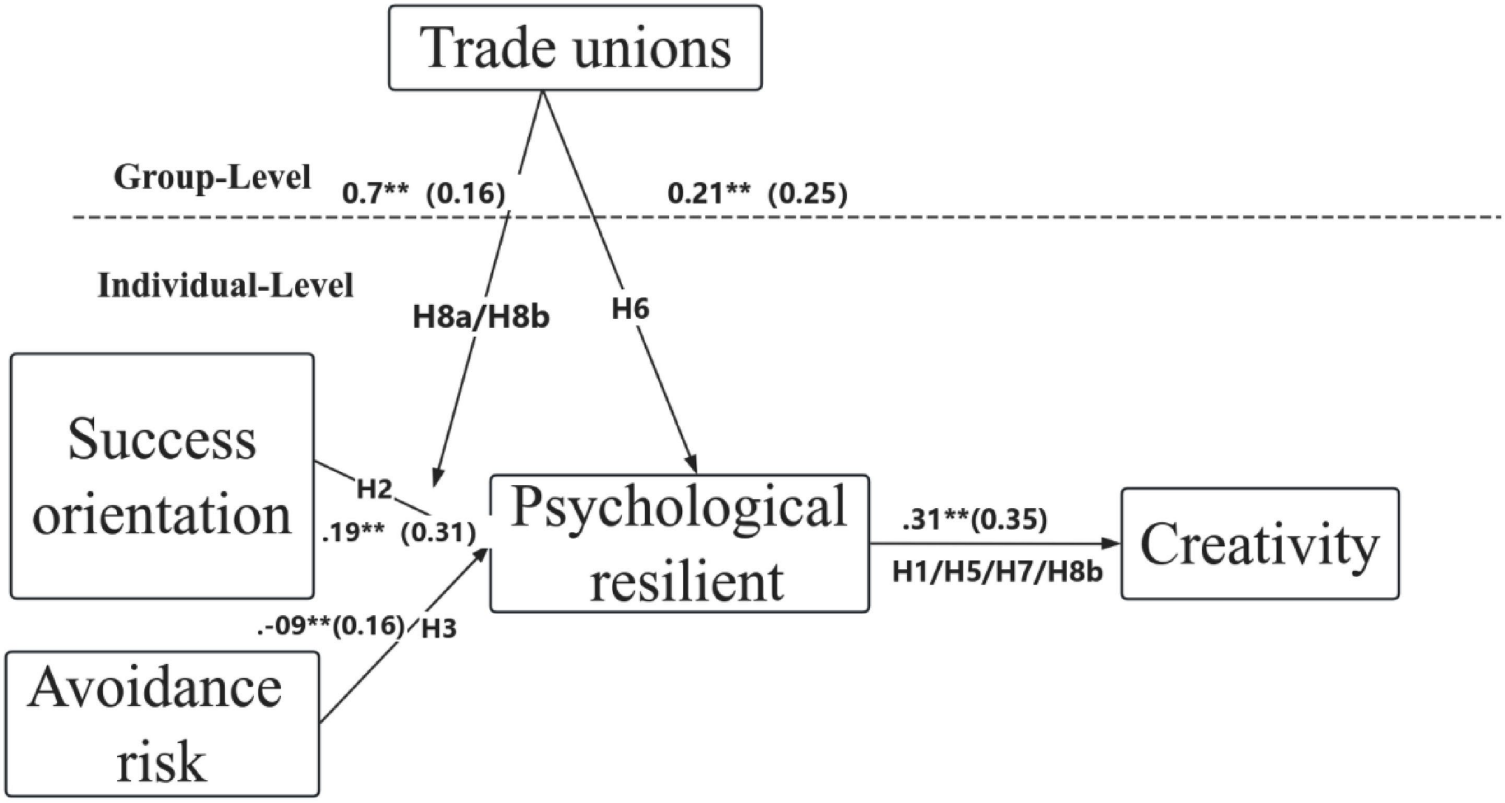
Path coefficients of the conditional process model. All path coefficients in the figure are standardized estimates (*β*). The cross-level moderating effect of employee unions was evaluated by calculating the difference in pseudo-R^2^ (ΔR^2^) between models with and without the interaction term. For simplicity of illustration, the paths from control variables (including age, gender, tenure, and educational level) to psychological resilience and creativity are not shown in the figure; complete results are available upon request. *p < 0.05, *p* < 0.01.

In the path model at the individual level, we also found significant random effects between success goal orientation and risk-avoidance orientation (Level 2, *p* < 0.05), suggesting that a moderator variable may need to be introduced to explain this variability (Bacharach et al., 2008). To this end, we followed the research method of Bauer et al. (2006) to further examine the multilevel moderated mediation effect. To assess cross-level relationships, we constructed a model that included both the direct association between employee participation climate and psychological resilience (main effect) and the random slopes of success goal orientation predicted by employee participation climate in relation to psychological resilience (cross-level moderating effect). In the supplementary analysis, we constructed a separate cross-level moderation model for risk-avoidance orientation to investigate whether the employee participation climate influences the relationship between risk-avoidance orientation and psychological resilience. The model results showed that employee participation climate had no significant effect on the random slope between risk-avoidance orientation and psychological resilience. Therefore, as expected, the cross-level moderating effect of risk-avoidance orientation was not supported. Next, we will focus on success goal orientation and discuss the estimated results obtained based on the proposed multilevel moderated mediation model (see Figure 2).

As shown in Figure 2, all relationships in the moderation-mediating model proposed in this study are statistically significant (*p* < 0.05). The results indicate that individual-level findings do not change significantly when employee participation climate is included in the analysis. Both success goal orientation and risk avoidance orientation are significantly associated with employee psychological resilience (*γ* = 0.19, *p* < 0.01; *γ* = −0.07, *p* < 0.01). Additionally, psychological resilience is significantly associated with creative behaviour (*γ* = 0.31, *p* < 0.01). We calculated the pseudo R^2^ (~*R*^2^) of the model using the formula proposed by Veall and Zimmermann (1996), which reflects the proportion of level-1 and level-2 errors reduced after including the predictor variables. The results show that the predictor variables explain 12.5% of the total variance in psychological resilience and 18.3% of the total variance in creative behaviour, indicating that success goal orientation, risk avoidance orientation, and employee participation climate have practical significance in predicting psychological resilience and creative behaviour.

The model results (Figure 2) support Hypotheses 6 and 7. The study demonstrates a significant positive correlation between employees’ perceived union support and psychological resilience (*γ* = 0.21, *p* < 0.01), with the indirect effect of psychological resilience on creativity through this pathway being 0.070. Using the Monte Carlo bootstrap method, the 95% confidence interval is 0.018–0.169.

**Figure 3:**
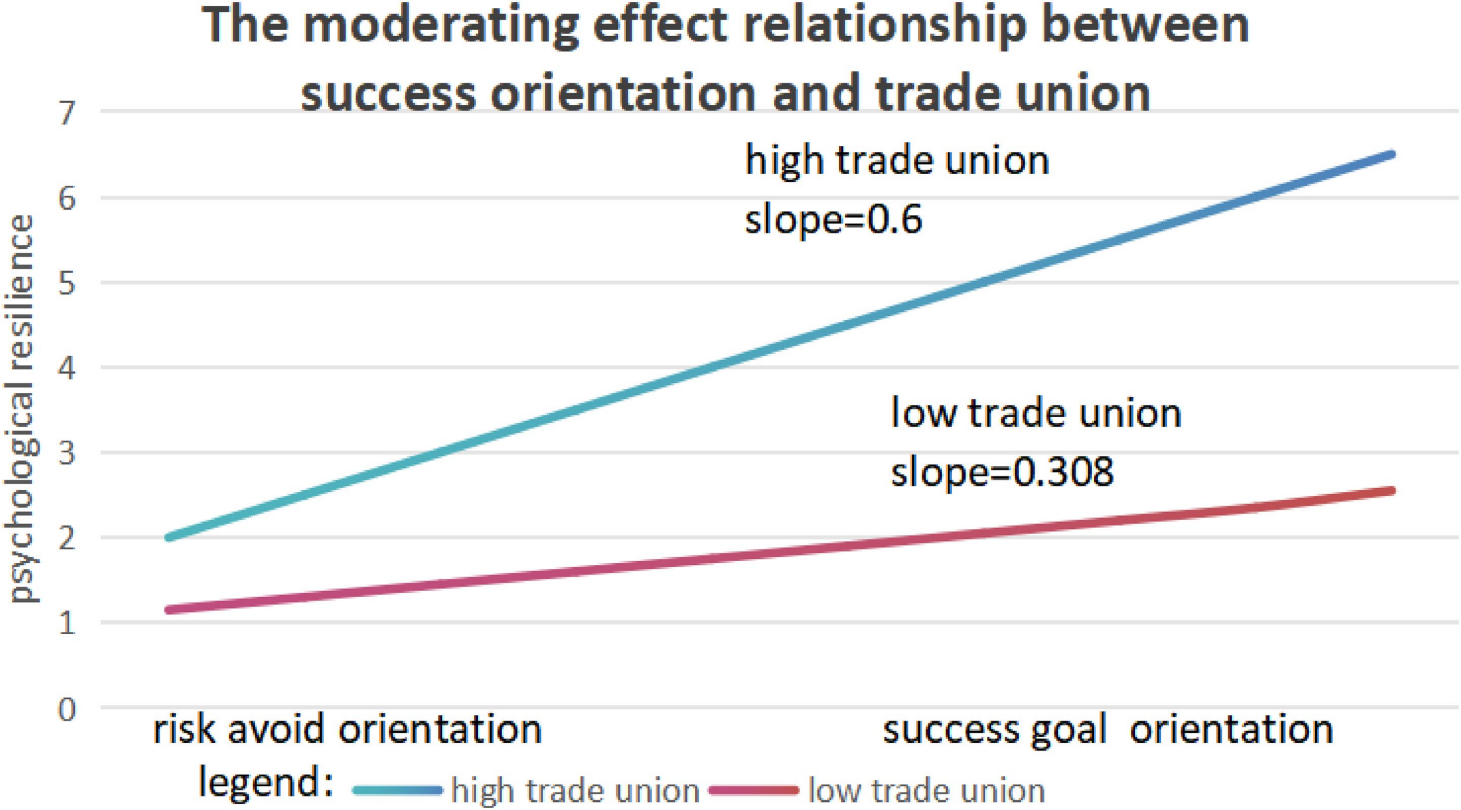
The moderating effect of employee trade union on the relationship between success orientation and psychological resilience.

Assuming hypothesis 8a predicts that the impact of goal-directed success orientation on employees’ psychological resilience will be moderated by the perceived union support atmosphere among employees. Multilevel model analysis results indicate that the perceived union support atmosphere has a positive moderating effect on the random slope between goal-directed success orientation and psychological resilience (*γ* = 0.07, *p* < 0.05). This phenomenon is typically referred to as a cross-level interaction, providing preliminary support for the ‘moderated mediation effect’ proposed in Hypothesis 8b (Chang et al., 2018). Following the research method of Tetrick et al. (2007), we plotted the interaction graph at two levels of perceived union support atmosphere: high (one standard deviation above the mean) and low (one standard deviation below the mean). As shown in Figure 3, when the perceived union support atmosphere is high, the positive association between goal-directed success orientation and psychological resilience is more pronounced than when the atmosphere is low. Therefore, Hypothesis 8a is validated.

Next, we employed the multilevel regression analysis method proposed byBauer et al. (2006) to determine the significance of the indirect effect of success goal orientation on creativity by evaluating performance in contexts with higher (+1 standard deviation) and lower (−1 standard deviation) levels of perceived employee union support atmosphere. The results presented in Table 2 show that the indirect effect of success goal orientation on creativity varies with changes in perceived employee union support: when perceived union support is higher, this indirect effect is more pronounced (estimate = 0.09, SE = 0.05, *p* < 0.05); whereas when the atmosphere is lower, the effect is weaker (estimate = 0.02, SE = 0.01, *p* > 0.05), which strongly supports Hypothesis 8b. Furthermore, when the perceived employee union support atmosphere is included as a moderator, the group-level residual variance in the slope of the relationship between success goal orientation and psychological resilience is no longer statistically significant ( σ2 = 0.02, *p* > 0.05). This indicates that the cross-level moderator significantly explains the group differences in this relationship, thereby explaining the variability in the indirect effect of success goal orientation on creativity through psychological resilience.

**Table 2:** Conditional indirect effects of success orientation on creativity through psychological resilience (divided by the level of trade union organizational strength).

Union organization strength level	Estimated indirect effect	Standard error (SE)	*p*-value
−1 standard deviation (low level)	0.02	0.01	0.156
Average value (medium level)	0.05	0.03	0.085
+1 standard deviation (high level)	0.09	0.05	0.032

*p < 0.05; **p < 0.01; ***p < 0.001.

In summary, the results of the multilevel path analysis fully support our proposed hypotheses regarding individual and organizational-level creativity processes, and also confirm the important role of perceived employee union support atmosphere in cross-level moderation.

## 5 Discussion and conclusion

The results of this study fully support the theoretical model constructed based on Social Cognitive Theory (SCT), confirming that employee creativity is a product of the complex interaction between individual traits and organizational environment through psychological mechanisms. Specifically, achievement goal orientation (including two dimensions: success orientation and risk aversion) and union participation climate both positively influence creativity by enhancing an individual’s psychological resilience, a key mediating variable. This not only confirms the core view of SCT regarding the triad reciprocal interaction among the individual, behaviour, and environment, but more importantly, it reveals that the union participation climate plays a crucial cross-level moderating role. In a supportive environment with a high degree of unionisation, the indirect promoting effect of success orientation on creativity through psychological resilience is significantly amplified, thereby enabling individuals with both high success orientation traits and a highly union-supported organizational environment to achieve the highest levels of creativity. This, on the one hand, clarifies the importance of psychological resilience as a core mediating mechanism, and on the other hand, highlights the key practical value of fostering a favourable climate for union participation to maximize employees’ innovative potential.

### 5.1 Theoretical implications

First, this study expands the theoretical framework of Social Cognitive Theory (SCT) by revealing how achievement goal orientation and union organizational atmosphere synergistically influence psychological resilience and creativity. Traditional organizational research has focused chiefly on two-dimensional relationships, neglecting the dynamic interaction mechanisms between individuals and their environment. Based on SCT, this paper constructs a multilevel, integrated model. It empirically tests the hypothesis that when individuals with a high achievement orientation are embedded in an organizational context with strong union support, their external relationships are optimized, and their autonomy and sense of competence are significantly enhanced. This effectively alleviates feelings of helplessness and anxiety, strengthens psychological resilience, and stimulates innovative behaviour. The research results show that unions not only function as resource providers and protectors of rights but also play the role of cross-level adaptive coordinators. By fostering a safe and supportive atmosphere, they activate individuals’ self-regulation mechanisms. It responds to Alagaraja and Shuck’s (2015) theoretical proposition regarding the alignment of individual and organisational goals, and further confirms that only when organizational structures (such as union participation) provide sufficient support can high achievement motivation be converted into actual innovative performance.

Second, this study clearly distinguishes the differential mechanisms by which success-oriented and risk-averse achievement goal orientations influence psychological resilience and creativity. Although both fall within the scope of achievement motivation, their psychological pathways are distinctly different. A success-oriented approach is characterized by proactive striving, sustained effort, and emotional vitality, which significantly and positively predict psychological resilience and creativity, serving as the ‘driving engine’ for innovative behaviour. In contrast, risk aversion manifests as a concern and avoidance of failure, which inhibits proactive exploration, weakens the development of resilience, and thereby negatively impacts creativity. This finding breaks through the limitations of previous research, which viewed achievement goal orientation as a single-dimensional construct, revealing its inherent heterogeneity and providing a more refined psychological mechanism to explain how motivation translates into innovative behaviour. Additionally, the study validates psychological resilience as a key mediating variable, highlighting its bridging role in connecting proactive goal pursuit with creative output, thereby enriching the application boundaries of achievement goal theory (Zusho and Clayton, 2011), in the context of organizational innovation.

Third, this study identifies psychological resilience as the core psychological mechanism linking individual traits and environmental factors in their joint influence on creativity. Through a three-stage time-lagged multilevel model analysis, we find that psychological resilience is not only an individual’s internal resource for coping with stress but also a dynamically constructed process influenced by the interactive effects of organizational support (such as union participation) and individual motivation (such as success orientation). Unions provide external support for resilience development by meeting needs for safety, belonging, and self-actualization; meanwhile, an individual’s achievement orientation determines whether they actively engage with and benefit from this support. This ‘individual-context’ interactive mechanism makes resilience a key mediating pathway for explaining differences in creativity. This study not only fills the gap in understanding how achievement goals influence innovative behaviour through psychological processes but also provides empirical evidence and practical guidance for organizations to cultivate employees’ resilience and unlock their innovative potential through institutional design (such as strengthening union functions).

### 5.2 Limitations and directions for future research

Firstly, the sample is limited to union organizations engaged in similar manufacturing tasks, with participants primarily holding non-managerial front-line positions. This restricts the generalisation of the research conclusions. Although the multitasking complexity of such positions provides a realistic basis for creative performance, and existing studies have confirmed the importance of creativity in low-cognitive-demand positions, the research findings still have difficulty being directly generalized to managerial positions or high-cognitive-oriented professions (such as R&D, strategic decision-making, etc.), which may have different motivational structures, levels of autonomy, and innovative contexts. Future research should expand sample diversity by including different industries, occupational levels, and organisational types to test the cross-situational robustness of the relationship between achievement goal orientation, psychological resilience, and creativity, and explore the moderating role of union system differences.

Secondly, although the three-stage lag design enhances the capability for causal inference, this study still falls under a quasi-longitudinal design, which fails to capture the dynamic evolution of psychological processes fully. This design cannot adequately reveal the bidirectional feedback mechanism between psychological resilience and creativity, for example, whether successful innovation experiences reverse enhance individual resilience through a resource-gain cycle (Hartwig et al., 2020). We should adopt accurate longitudinal tracking or intensive experience sampling methods (ESM) to observe individuals’ emotional fluctuations, behavioural adjustments, and changes in psychological resources within the creativity cycle over a longer time span, in order to test whether resilience is strengthened after creative achievements and further influences subsequent innovation motivation and stress resistance.

Finally, this study has integrated individual and situational factors; however, there may still be important variables that have not been incorporated and affect the complete explanation of differences in creativity. For example, at the individual level, types of motivation and creative personality traits; at the team level, cohesion and knowledge sharing; at the organizational level, cultural support and leadership styles (such as goal-oriented and participatory leadership); and in the external environment, organizational relationships (such as customer cooperation) and competitive pressure may all moderate or mediate the core pathways. In addition, the cognitive load brought by off-work demands (such as family responsibilities) may also weaken the positive role of success orientation. We should construct a more comprehensive, multilevel model that systematically incorporates these variables and explores how individual, team, organizational, and environmental factors jointly shape the innovation process within a trade union participation context.

### 5.3 Practical implications and conclusion

First, managers should regard enhancing employees’ psychological resilience as a core strategy to stimulate creativity. This study demonstrates that psychological resilience serves as a key mediating factor between achievement goal orientation and creativity. The resilience, adaptability, and sustained commitment employees demonstrate when facing challenges form the psychological foundation for their creative work. Therefore, organizations should systematically enhance employees’ psychological resilience through means such as providing emotional support, stress management training, and growth-oriented feedback mechanisms, thereby providing continuous motivation for innovative behaviour.

Second, the trade union participation atmosphere can play a constructive role in enhancing psychological resilience and promoting creativity by providing resource support and creating a positive environment. The trade union participation atmosphere is not only a representative of rights and interests but also an important cultivator of organizational psychological capital. By promoting highly participatory governance, strengthening informal relationship networks, and facilitating knowledge sharing and collective learning, the trade union participation atmosphere can effectively alleviate individuals’ sense of helplessness and anxiety, and enhance members’ autonomy and sense of belonging. This supportive environment provides external guarantees for the development of employees’ psychological resilience, thereby activating their innovative potential and achieving a transformation from “defensive coping” to “active creation.”

Thirdly, enterprises should combine goal-oriented talent selection with the creation of a supportive work environment to maximize innovative outcomes. Neither individual traits nor organizational mechanisms alone are sufficient to drive creativity; true innovation stems from the synergistic effect of ‘people’ and ‘environment’. In recruitment and allocation, organisations should prioritise employees with success-oriented traits, while strengthening the functions of unions through institutional design, thereby creating a bridge that connects individual motivations with organisational objectives. Only by embedding individuals with high achievement motivation into a supportive and inclusive union culture can a virtuous cycle of psychological resilience and creativity be achieved, thereby building a sustainable competitive advantage in innovation.

## Data Availability

The raw data supporting the conclusions of this article will be made available by the authors, without undue reservation.
